# Advances in immunotherapy for HPV-associated malignancies: emerging strategies and clinical progress

**DOI:** 10.3389/fimmu.2026.1897025

**Published:** 2026-09-16

**Authors:** Alaa Alsalloum, Alena Zakhareva, Julia Lopatnikova, Sergey Sennikov

**Affiliations:** 1 Laboratory of Molecular Immunology, Federal State Budgetary Scientific Institution Research Institute of Fundamental and Clinical Immunology, Novosibirsk, Russia; 2 Department of Natural Sciences, Novosibirsk State University, Novosibirsk, Russia; 3 Federal State Autonomous Educational Institution of Higher Education, I.M. Sechenov First Moscow State Medical University of the Ministry of Health of the Russian Federation, Sechenov University, Moscow, Russia; 4 Department of Immunology, Institute of Medicine and Medical Technologies, Novosibirsk State University, Novosibirsk, Russia

**Keywords:** human papillomavirus, HPV, E6 and E7, immune response, therapeutic vaccine, Engineered TCR T cells

## Abstract

Human papillomavirus (HPV) is the principal etiological factor in the majority of cervical, oropharyngeal, anal, and vulvar cancers. Persistent infection with high-risk HPV types promotes immune evasion, malignant transformation, and tumor progression. Currently licensed prophylactic HPV vaccines, based on L1 virus-like particles, are highly effective at preventing new infections and HPV-associated neoplastic disease through the induction of neutralizing antibodies. However, they do not eradicate established infections or preexisting lesions. These limitations have driven the development of therapeutic HPV vaccines and adoptive T-cell strategies aimed at eliciting robust cell-mediated immunity against HPV-associated malignancies. The viral oncoproteins E6 and E7 are attractive therapeutic targets. They disrupt cell-cycle control and are consistently expressed in HPV-driven precancerous and cancerous lesions. In this review, we provide a comprehensive overview of therapeutic HPV vaccines—including viral and bacterial vectors, DNA and RNA constructs, peptide/protein vaccines, and cell-based approaches—all designed to target E6 and E7, while considering the benefits and limitations of each platform. We also present an in-depth analysis of adoptive T-cell therapies, highlighting Immuno-STAT, a peptide–HLA fusion platform for the direct expansion of HPV-specific CD8^+^ T cells. Furthermore, we discuss rational combination strategies that integrate these modalities with immune checkpoint inhibitors and other immunomodulatory agents, including the bifunctional PD-L1/TGF-β inhibitor bintrafusp alfa, to overcome key barriers and enhance clinical efficacy. Overall, this review synthesizes current evidence and outlines future directions for the development of more precisely tailored HPV-specific immunotherapies in cancer treatment.

## 1 Introduction

Human papillomavirus (HPV) is the most widespread sexually transmitted pathogen globally and continues to pose a substantial public health burden. Epidemiological studies estimate that over 80% of sexually active individuals will contract genital HPV at least once by the age of 50 (1, 2). Although most infections are transient and resolve spontaneously, 10–20% persist and carry a risk of progressing to premalignant or invasive lesions (3). Approximately 90% of cervical and anal cancers, 70% of vulvar and vaginal cancers, 60% of penile cancers, and 70% of oropharyngeal cancers are attributable to HPV infection (4).

The etiological link between HPV and cervical cancer was first proposed by Harald zur Hausen in the 1970s. Within a decade, his group isolated HPV16 and HPV18 from cervical carcinoma biopsies—discoveries that fundamentally reshaped cancer biology and were later recognized with the Nobel Prize in Medicine (5). To date, more than 229 HPV genotypes have been identified and classified into low-risk and high-risk groups based on their oncogenic potential (6–8). Low-risk types, such as HPV6 and HPV11, are typically associated with benign lesions, including anogenital warts and recurrent respiratory papillomatosis (9, 10). In contrast, high-risk HPV types, particularly HPV16 and HPV18, are the principal oncogenic drivers of HPV-associated malignancies. HPV16 accounts for approximately 50% of cervical cancer cases, while HPV18 contributes to about 20% (11). Notably, HPV16 is also responsible for nearly 90% of HPV-positive oropharyngeal carcinomas (12, 13).

HPV is a small, circular, double-stranded DNA virus of the Papillomaviridae family, with a genome of approximately 8 kb (**Figure 1**). The genome encodes early proteins (E1–E8), which regulate viral replication, transcription, and host-cell proliferation, and late structural proteins (L1 and L2), which assemble the viral capsid (14–16). In persistent infections, the viral genome may integrate into host chromosomal DNA—a hallmark of high-risk HPV–associated neoplasia. Integration frequently disrupts the E1/E2 region, leading to loss of E2-mediated repression of viral transcription and sustained overexpression of the viral oncogenes E6 and E7, a key event in malignant transformation (17–19).

**Figure 1:**
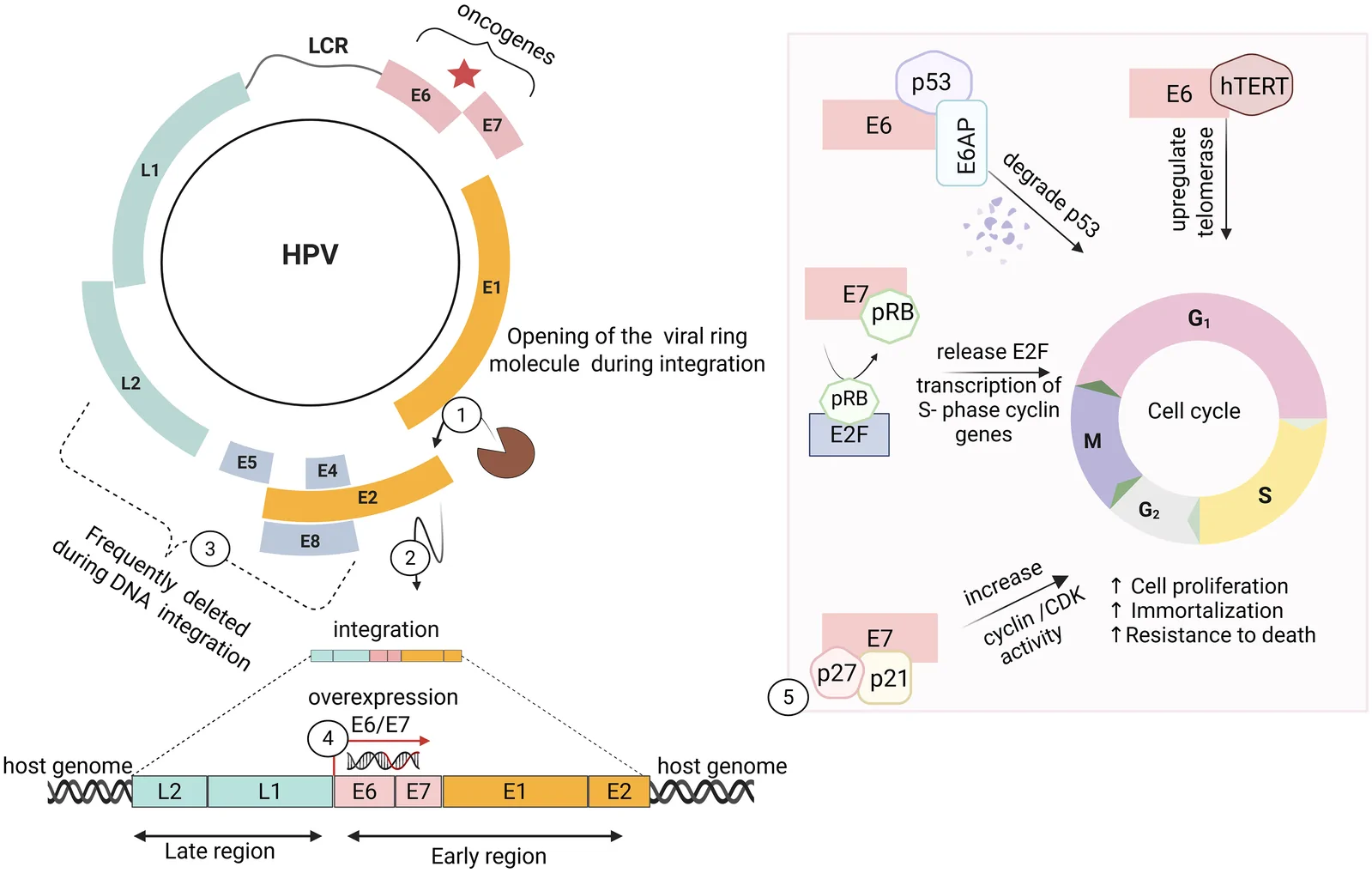
Genomic organization of human papillomavirus (HPV) and molecular events associated with malignant progression. The HPV genome is a circular double-stranded DNA molecule composed of an early region containing the E1, E2, E4, E5, E6, E7, and E8 genes; a late region encoding the major and minor capsid proteins L1 and L2; and a long control region (LCR). Early viral proteins regulate genome replication, transcription, and modulation of host cellular processes. During persistent infection, HPV DNA can integrate into the host genome, frequently involving disruption of the E2 region. Integration may also result in deletion of variable adjacent viral sequences, leading to loss of E2-mediated transcriptional repression and subsequent overexpression of the viral oncoproteins E6 and E7, which interfere with key cellular checkpoints and promote malignant progression.

The E6 and E7 oncoproteins target central tumor suppressor pathways (**Figure 1**). E7 binds to and functionally inactivates the retinoblastoma protein (pRB) through ubiquitin-mediated degradation, resulting in the release of E2F transcription factors and induction of S-phase cyclin genes (cyclins A and E), thereby promoting aberrant G1–S cell-cycle progression (20–23). E7 also disrupts the cyclin-dependent kinase inhibitors p21 and p27, further enhancing CDK2-dependent proliferation (24, 25). E6 recruits the E3 ubiquitin ligase E6AP to target p53 for proteasomal degradation, suppressing apoptosis and enabling survival of genetically unstable cells (26–29). Beyond these canonical effects, E6 targets PDZ-domain proteins involved in epithelial polarity and cell–cell adhesion, contributing to tissue disorganization and hyperplasia (30–32). E6 further promotes cellular immortalization through upregulation of the telomerase catalytic subunit hTERT and interactions with telomeric components (33, 34). In addition, the HPV E5 protein, particularly from HPV16, has been implicated in early stages of carcinogenesis, where it may contribute to growth factor signaling and immune evasion (35, 36).

Recognition of HPV as the causal agent of cervical and other HPV-associated malignancies has enabled major advances in cancer prevention and therapeutic development. Three prophylactic HPV vaccines are currently licensed; all are based on L1 virus-like particles that induce neutralizing antibodies, block viral entry, and provide robust protection against the most oncogenic HPV types (37–39). However, these vaccines do not treat established infections or pre-existing lesions, as L1 capsid proteins are not expressed in infected basal epithelial cells (37–39). However, these vaccines do not treat established infections or pre-existing lesions, as L1 capsid proteins are not expressed in infected basal epithelial cells (40), underscoring the need for therapeutic strategies capable of eliminating HPV-infected or transformed cells. Accordingly, increasing attention has focused on therapeutic HPV vaccines that elicit cell-mediated immune responses. These strategies primarily focus on the viral oncoproteins E6 and E7, which are indispensable for tumor initiation and maintenance and are constitutively expressed in HPV-driven precancerous and malignant cells. Although E5 has also been investigated as an immunotherapeutic target, its expression is frequently reduced or lost following viral genome integration, limiting its suitability as a therapeutic target in established HPV-associated malignancies (41). Because E6 and E7 are viral antigens absent from normal host tissues, they largely avoid central immune tolerance, making them particularly attractive targets for immunotherapy (42). A broad spectrum of therapeutic platforms has been developed to deliver E6/E7-derived antigens to antigen-presenting cells and induce robust cytotoxic T lymphocyte responses. In this review, we summarize current advances in therapeutic HPV vaccines and T cell–based immunotherapies, examine the integration of immune checkpoint blockade into HPV-directed treatment strategies, and highlight ongoing challenges and future directions toward improving immunological control of HPV-associated diseases.

## 2 The immune response against HPV infection

Innate and adaptive immune responses are central to the control of HPV infection; however, inter-individual variability in the specificity and magnitude of these responses may permit sequential or concurrent infection with multiple HPV genotypes. Consequently, clinical outcome is determined by both viral virulence and the effectiveness of host immunity (43). In most individuals, high-risk HPV infection is eventually cleared through T-cell-mediated recognition of viral antigens. However, in approximately 10–15% of cases, these responses are insufficient, resulting in persistent infection and the development of epithelial lesions that may persist for years (44).

HPV persistence is promoted by multiple immune-evasion mechanisms. The virus maintains extremely low levels of gene expression (10–100 copies per cell) within epithelial cells, thereby limiting major histocompatibility complex (MHC) class I-mediated antigen presentation to cytotoxic T lymphocytes. In addition, depletion of Langerhans cells within infected epithelia impairs antigen uptake and T-cell priming, further weakening local immune surveillance (45–48).

Immune recognition also varies according to disease stage. E2- and E6-derived antigens are preferentially recognized in low-grade lesions, whereas E7 epitopes predominate in T-cell responses associated with high-grade disease (49, 50). The composition of the immune microenvironment is also influenced by HPV integration status; integration-negative tumors exhibit enriched immune-cell signatures associated with CD4^+^ T cells, CD8^+^ T cells, regulatory T cells, NK cells and B cells, correlating with more favorable clinical outcomes than integration-positive tumors (51).

Persistent infection is further sustained by immunomodulatory activities of viral oncoproteins. E6 and E7 interfere with NF-κB signaling and suppress type I interferon responses, whereas E5 contributes to immune escape through downregulation of MHC class I and CD1d molecules, thereby limiting recognition by cytotoxic T lymphocytes and NK cells (52, 53).

In parallel, increased infiltration of regulatory T cells, together with elevated production of immunosuppressive cytokines, including IL-10 and transforming growth factor-β (TGF-β), promotes a tolerogenic microenvironment that impairs effective viral clearance. Collectively, these mechanisms sustain viral oncogene expression and drive the progressive transition from persistent infection to dysplasia and ultimately invasive HPV-driven malignancy (43, 48, 54, 55).

## 3 Therapeutic HPV vaccines

Progress in understanding HPV-driven oncogenesis and the identification of immunogenic viral epitopes have enabled the development of immunotherapeutic strategies targeting HPV-associated malignancies. Therapeutic vaccines aim to induce antigen-specific T-cell responses capable of eliminating HPV-infected or transformed cells, in contrast to prophylactic vaccines, which prevent infection through the induction of neutralizing antibodies (56, 57). Current therapeutic vaccine strategies include peptide- and protein-based vaccines, viral vector vaccines, DNA and RNA vaccines, and cell–based approaches (**Figure 2**). Although no therapeutic HPV vaccine has yet been approved for clinical use, numerous candidates have demonstrated promising immunogenicity and antitumor activity in preclinical and clinical studies (58–60). Therapeutic immunotherapy strategies targeting HPV-associated malignancies and their clinical evaluation are summarized in **Table 1**.

**Figure 2:**
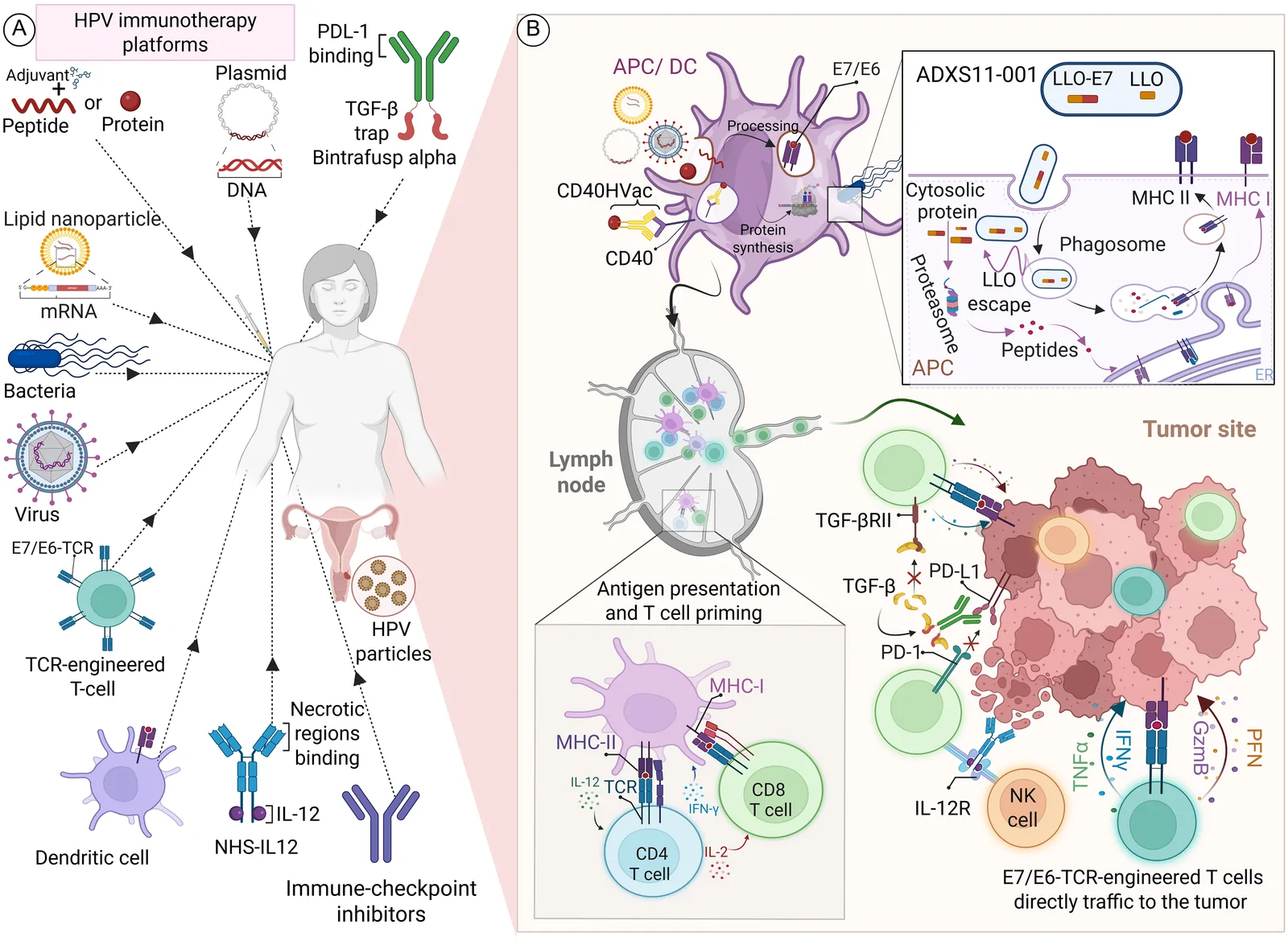
Therapeutic immunotherapy strategies targeting HPV-associated malignancies: platforms **(A)** and mechanisms **(B)**. Following administration of therapeutic HPV immunotherapies, HPV E6/E7 antigens are captured by antigen-presenting cells (APCs), predominantly immature dendritic cells, which undergo maturation and migrate to the draining lymph node. There, processed antigens are presented through MHC class I and II pathways, leading to priming and activation of HPV-specific CD8⁺ cytotoxic and CD4⁺ helper T-cell responses. CD40HVac enhances this process by targeting dendritic cells through CD40-mediated delivery of HPV16 antigens. The Listeria monocytogenes-based vaccine ADXS11-001 expresses HPV16 E7 fused to a non-hemolytic fragment of listeriolysin O (LLO), facilitating phagosomal escape and cytosolic antigen processing to enhance MHC class I presentation. Activated effector T cells subsequently traffic to the tumor site, where they recognize HPV antigen-expressing tumor cells and mediate cytotoxic elimination through perforin-, granzyme B-, and IFN-γ-dependent mechanisms. In contrast, adoptively transferred HPV-specific TCR-engineered T cells directly migrate to the tumor site following infusion. At the tumor site, Bintrafusp alfa simultaneously blocks PD-L1 signaling and sequesters TGF-β to reduce immunosuppressive signaling. NHS-IL12, a tumor-targeting antibody fused to IL-12, promotes IL-12 receptor-mediated activation of T cells and natural killer (NK) cells, thereby enhancing antitumor immunity.

**Table 1:** Overview of clinical trials of therapeutic immunotherapy strategies targeting HPV-associated malignancies.

Identifier	Phase	Tumor type	Therapeutic platform	Epitope	Key findings n/N (%)
NCT02426892 (66, 70, 193)	II	Incurable HPV16+ cancers (mostly oropharyngeal)	ISA101 (synthetic long overlapping peptides vaccine in Montanide ISA-51) combined with nivolumab (anti-PD-1)	13 SLPs covering full HPV16 E6 and E7 proteins: 9 overlapping E6 peptides (five 32-mers, four 25-mers) and 4 overlapping 35-mer E7 peptides	• ORR: 33% • mDOR: 11.2 months • mOS: 15.3 months • Higher tumor infiltration CD3+CD8+PD-1+ cytotoxic T cells and CD68+PD-L1− and CD68+PD-L1+ macrophages correlated with better clinical response • Responders had higher expression of immune, inflammatory, and interferon-signaling genes • AEs included asymptomatic grade 3 transaminase elevation and grade 4 lipase elevation leading to nivolumab discontinuation
NCT03669718 (194)	II	Recurrent/metastatic HPV16+ oropharyngeal cancer	ISA101b (peltopepimut-S) synthetic long overlapping peptides vaccine combined with cemiplimab (anti-PD-1) versus cemiplimab alone	12 SLPs derived from HPV16: 9 overlapping E6 peptides (identical to ISA101) and 3 overlapping 35-mer E7 peptides	• Adding ISA101 to cemiplimab did not improve ORR and worsened mOS • Patients with high PD-L1 combined positive score (CPS ≥20) had higher ORR and longer OS • Benefits limited to high PD-L1 expressers • No increase in toxicity from adding ISA101b
NCT03821272 (195)	II	HPV16+ head and neck squamous cell carcinoma (HNSCC)	PepCan: mixture of synthetic peptides mixed with a Candida skin testing reagent (Candin^®^)	Four synthetic peptides covering the entire HPV16 E6 protein (E6 _1–45_, E6 _46–80_, E6 _81–115_, and E6 _116–158_)	• Generally safe • The vaccine did not significantly reduce cancer recurrence compared to placebo, but the results are inconclusive due to the very small number of patients (17 patients) • Induced T-cell immune responses against HPV16 E6 in some patients
NCT02481414 (196)	II	cervical intraepithelial neoplasia (CIN)2/3	PepCan OR Candan^®^		T-cell responses to HPV16 E6: • 54.5% (PepCan group) • 44.7% (Candin group)
NCT02821494 (70)	I	HPV16+ (pre)malignant lesions	Hespecta: synthetic long peptides coupled to Amplivant, a synthetic TLR2 ligand	Two SLPs of derived from HPV16 E6 (E6 _71–95_ and E6 _127–158_)	• Generally safe with only mild grade 1/2 • Clear dose–response • Induced durable HPV16-specific T-cell responses
NCT04260126 (197)	II	Recurrent/metastatic HPV16+ HNSCC	PDS0101: short-peptide HPV16 vaccine in an R-DOTAP liposomal carrier, which serves as adjuvant combined with pembrolizumab (anti-PD-1 antibody)	six HLA-unrestricted HPV16 E6 and E7 peptides	• Well tolerated • ORR: from 26.5% to 38.9%, including 1 CR, 9 PR and 15 SD • mPFS: 10.4 months • The estimated 12-month OS rate: 87.1%, with mOS not reached at data cutoff • TRAEs to PDS0101: 68.5%, mostly grade 1/2
NCT04287868 (72)	II	Advanced/metastatic HPV16+ cancers including, ICB-naïve and resistant patient	Combination immunotherapy: PDS0101 + PDS01ADC (IL-12 antibody-drug conjugate) + bintrafusp alfa (PD-L1/TGF-β inhibitor)		Among patients with HPV-16-positive tumors • ORR: 62.5% • mOS not reached • Grade 3/4 TRAEs occurred in about half • No treatment-related deaths
NCT06007092 (198)	I/IIa	HPV16+ oropharyngeal carcinoma (OPC)	CD40HVac: HPV16 proteins fused to a humanized IgG4 monoclonal antibody targeting CD40 receptor of DC, adjuvanted with poly-ICLC	HPV16 E6/E7 proteins	• Preliminary data demonstrated an acceptable safety profile • Induced functional HPV16 E6/E7-specific CD4+ and CD8+ T-cell responses
NCT01304524 (94)	II	CIN 2/3	VGX-3100: DNA vaccine with two plasmids encoding optimized synthetic consensus E6/E7 genes from HPV16 and HPV18, delivered IM followed by electroporation	HPV16/18 E6 and E7	• Well tolerated: only injection site erythema common but no serious adverse events Histopathological regression: • 49.5% (53/107) VGX-3100 • 30.6% (11/36) placebo Concurrent regression plus HPV16/18 clearance: • 40.2% (43/107) VSX-3100 • 14.3% (5/35) placebo
NCT02139267 (91)	II	CIN3	GX-188E: DNA vaccine encoding shuffled fusion protein of HPV16/18 E6/E7 fused to Flt3L domain for DC targeting and tPA signal sequence for secretion; delivered IM with electroporation	HPV16/18 E6 and E7	• Histopathologic regression: 52% (33/64) at 20 weeks; 67% (35/52) at 36 weeks post first injection • HPV clearance significantly associated with regression • Patients with HPV clearance in 36 weeks post first injection showed higher IFNγ responses compared with those without HPV clearance • Variants sequence of HPV16 E6/E7 (D25E, V83L, and N29S) inversely associated with regression
NCT03444376 (199)	II	Advanced HPV16+/18+ cervical cancer	GX-188E combined with pembrolizumab (anti-PD-1)		• ORR: 35% (21/60), including 5 CR and 16 PR • mPFS: 4.4 months • mOS: 23.8 months • TRAEs of any grade: 33.8% including 4 patients in grade 3/4
NCT04405349 (200, 201)	IIa	Persistent, recurrent, or metastatic HPV16+ cervical cancer	VB10.16: DNA vaccine encoding mutation-inactivated HPV16 E6 and E7 oncoproteins fused to CCL3L1 chemokine ligand for targeting antigen-presenting cells, combined with atezolizumab (anti-PD-L1)	HPV16 E6/E7	• Well tolerated • ORR: 19.1% overall; 29.2% in PD-L1+ patients • mPFS: 4.1 months • mOS: 21.3 months • HPV16-specific T-cell responses in 61% of evaluable patients • Early circulating tumor DNA reduction correlated with clinical benefit
NCT02172911 (95)	I/IIa	Persistent/recurrent HPV16+/18+ cervical cancer	MEDI0457 (INO-3112): combination of VGX-3100 plasmids plus IL-12 encoding plasmid to enhance cellular immunity; IM electroporation delivery	HPV16/18 E6 and E7	• Well tolerated with only grade 1 adverse events mostly at injection sites • 8/10 patients developed cellular or humoral immune responses (7 CR, 1 PR) • Cervical biopsies showed decreased PD-1^+^CD8^+^, PD-L1^+^CD8^+^, and PD-L1^+^CD68^+^ subpopulations with no HPV DNA
NCT03162224 (202)	Ib/IIa	Recurrent/metastatic HPV16+/18+ HNSCC	MEDI0457 combined with durvalumab (anti-PD-L1)		Among 29 evaluable patients • ORR: 27.6% including 4 CR and 4 PR • Responses were independent of PD-L1 tumor expression level • mPFS: 3.5 months • mOS: 29.2 months • TRAEs: 80%, 5 patients in grade 3 resulting in discontinuation in 2 patients
NCT00988559 (92)	I	CIN2/3	pNGVL4a-CRT/E7(detox): DNA plasmid expressing mutated HPV16 E7 fused to calreticulin (CRT) to enhance MHC class I presentation; administered by PMED, IM, or cervical intralesional injection	HPV16 E7 (mutated at aa24/27)	• Histologic regression: 30% (8/27); one patient regressed to CIN1, 7 no residual CIN • Elicited robust immune response especially when administered intralesionally • No differences between pre-and post-vaccination viral loads, in any of the treatment cohorts
NCT00121173 (93)	I	CIN2/3	pNGVL4a-Sig/E7(detox)/HSP70: DNA plasmid expressing mutated HPV16 E7 fused to a signal sequence (Sig) and HSP70 for enhanced MHC class II presentation and CD4+ T cell activation; IM injection	HPV16 E7 (mutated at aa24/27)	• Well tolerated • 3/9 had complete histologic regression of CIN2/3 in the highest dose cohort • Low but detectable increases in HPV E7-specific T-cell responses
NCT04534205 (105)	II	Recurrent or metastatic PD-L1+ HNSCC	BNT113: mRNA-lipoplex (LPX) combined with pembrolizumab	HPV16 E6/E7	• Well tolerated • mOS: 22.6 months • mPFS: 3.9 months • 6/15 patients responded including 4 CR, 2 PR and 2 SD • Induced T-cell responses against HPV16 E6 and E7
NCT01266460 (203)	II	Platinum-refractory cervical carcinoma	ADXS11-001: attenuated Listeria monocytogenes bacterial vector expressing tLLO-HPV-16 E7 fusion protein	full-length of HPV-16 E7	• Well tolerated; the majority of TRAEs were grade 1/2 • The 12-month OS: 38% (19/50) • mOS: 6.1 months • mPFS: 2.8 months
UMIN000001686 (127)	I/IIa	CIN3	GLBL101c: attenuated Lactobacillus casei bacterial vector expressing modified full-length HPV16 E7 protein	HPV16 E7 mutated into the Rb-binding site (the D, C, and E in E7 aa21, aa24, and aa26, respectively, were all replaced by a G)	• 70% of patients showed pathological regression from CIN3 to CIN2 • No adverse events • Induced significant E7-specific mucosal IFN-γ–producing T cells in cervical lymphocytes • This study is the first to demonstrate a direct correlation between mucosal E7-specific cellular immunity and clinical response in human mucosal neoplasia
NCT03260023 (129)	Ib/II	Advanced/recurrent HPV16^+^ cancers (mainly anal, plus oropharyngeal, cervical, vulvar/vaginal)	TG4001: Viral vector vaccine (attenuated vaccinia virus Ankara expressing modified HPV16 E6/E7 and IL-2), combined with avelumab (anti-PD-L1)	HPV16 E6 and E7	• No dose-limiting toxicities • TRAEs: 93%, mostly grade 1–2; one grade 3 anemia • ORR: 22% (8/36) overall • mPFS: 2.8 months • mOS: 11.0 months overall • Antigen-specific T-cell responses detected in 7/11 patients
NCT03141463 (140)	I/II	HPV16+ CIN3	Vvax001: Viral vector vaccine (a replication-incompetent Semliki Forest virus expressing HPV16 E6/E7)	HPV16 E6 and E7	• Well tolerated • Histologic complete response (≤ CIN1 or no dysplasia): 50% (9/18) • HPV16 clearance: 63% (10/16) • mDFS: 20 months
NCT04180215 (141)	I/II	HPV16+ recurrent or metastatic HNSCC	HB-201 and HB-202: replication-competent non-lytic arenavirus vectors (HB-201: lymphocytic choriomeningitis virus; HB-202: Pichinde virus), given as single vector or alternating HB-201/HB-202	HPV16 E6 and E7	• HB-201 or HB-202 monotherapy is highly immunogenic, with marked inflammatory cytokine/chemokine induction and expansion of HPV16 E6/E7-specific CD8^+^ T cells • Alternating HB-202/HB-201 dosing increased HPV16-specific CD8^+^ T cells to up to 40% of total peripheral CD8^+^ T cells Among 11 evaluable HPV^+^ HNSCC patients treated with HB-201: • ORR: 18%, including 2 PR • DCR: 73% • mPFS: 3.45 months
NCT02866006 (155, 156)	IIa	Persistent/recurrent HPV16+/18+ cervical cancer	BVAC-C: autologous B cells and monocytes transfected with HPV E6/E7 recombinant genes and loaded with α-galactosylceramide (NKT ligand); intravenously as a monotherapy and in combination with topotecan (chemotherapy drug)	HPV16/18 E6/E7	Among 30 evaluable patients: • ORR: 19.2% • DCR: 53.8% • mDOR: 7.5 months • mPFS: 5.8 months • mOS: 17.7 months • Induced the activation of NKT cells, NK cells, and E6/E7-specific T cells • Well tolerated • Topotecan did not improve clinical outcomes compared with BVAC-C monotherapy
NCT03978689 (175–177)	I	Recurrent/metastatic HPV16+ HNSCC	CUE-101: Immuno-STAT fusion protein composed of 2 peptide-HLA complexes (HLA-A*0201/E7 peptide), 4 reduced affinity IL-2 molecules, and effector attenuated IgG1 Fc domain; used alone or with pembrolizumab	HPV16 E7_11-20_ (YMLDLQPETT)	• Well tolerated • Monotherapy showed 1 PR, 6 SD ≥12 weeks; mOS: 20.8 months; induced HPV16 E7_11-20_-specific CD8+ T cells, NK cells and transient increase in regulatory T cells (Tregs) • Combination therapy showed ORR 47% (1 CR, 8 PRs), mPFS 5.8 months, mOS not reached, and >99% drop in circulating HPV16 DNA fragments in plasma
NCT01585428 (160)	I/II	Metastatic or refractory/recurrent cervical cancer	Young TIL therapy	HPV16/18 E6/E7	• OR: 3/9 patients (2 CR, 1 PR) • The CR remained ongoing at 22 and 15 months, and the PR lasted 3 months • The HPV reactivity of T cells in the infusion product correlated positively with clinical response
NCT02280811 (163)	I/II	Metastatic or refractory/recurrent HPV16+ cancers (cervical SCC, cervical adenocarcinoma, oropharyngeal SCC, vaginal SCC, anal SCC)	Genetically TCR-engineered T cells; Cyclophosphamid fludarabine; aldesleukin	HPV16 E6_29–38_/HLA-A*02:01 (TIHDIILECV)	• Safe, feasible, and generally manageable • OR: 2/12 patients (both anal SCC); one patient remained disease-free 3 years after treatment • At ~1 month, E6 TCR T cells were a median 30% (4–53%) of circulating CD3^+^ T cells and retained functional reactivity to the E6 peptide • Resistance mechanisms included IFNGR1 frameshift mutation and loss of HLA-A*02:01
NCT02858310 (166)	I	Metastatic or refractory/recurrent HPV-16+ cancers (cervical SCC, anal SCC, vulvar SCC, cervical adenocarcinoma, head and neck SCC)	Genetically TCR-engineered T cells; Cyclophosphamid and aldesleukin	HPV 16 E7_11–19/_HLA-A*02:01 (YMLDLQPET)	• Safe and generally well tolerated • OR: 6/12 patients, including 4 of 8 with anti–PD-1–refractory disease • Three patients demonstrated complete regression of ≥1 metastatic lesion, including extensive regression of ~25 and ~80 metastases, with responses lasting 8–14 months • Tumor resistance was associated with defects in antigen processing and interferon signaling pathways, including copy-number loss of TAP1/2 and IFNGR1/2, biallelic loss of B2M, and mutations in HLA-A*02:01
NCT05686226	II				Recruiting
NCT06358053	I	advanced HPV16 + cancers	CRTE7A2–01 TCR-T Cells: genetically TCR-engineered T cells	E7/HLA-A*02:01	Not yet recruiting

Values are expressed as the percentage of patients, % (n/N), for the indicated endpoints.

SLPs, synthetic long peptides; ORR, overall response rate; mDOR, median duration of response; mOS, median overall survival; CR, complete response; PR, partial response; mDFS, median disease-free survival; DCR, disease control rate; mPFS, median progression-free survival; SCC, squamous cell carcinoma; IM, intramuscular; PMED, particle-mediated epidermal delivery; DC, dendritic cell; HLA, human leukocyte antigen; NK, natural killer; tLLO, truncated listeriolysin O; OR, objective response; PD-1, programmed cell death protein 1; TRAEs, treatment-related adverse events; TCR, T-cell receptor; TIL, tumor-infiltrating lymphocyte; poly ICLC, polyinosinic-polycytidylic acid stabilized with poly-L-lysine and carboxymethylcellulose; Immuno-STAT, selective targeting and alteration of T cells; TPA, tissue plasminogen activator; Flt3L, FMS-like tyrosine kinase 3 ligand. Statements

### 3.1 Peptide- and protein-based vaccines

Peptide- and protein-based vaccines targeting human papillomavirus (HPV) represent a key strategy in the development of therapeutic cancer vaccines, aimed at inducing antigen-specific T-cell responses against viral oncoproteins. Peptide vaccines are particularly attractive owing to their safety, specificity and ease of manufacture; however, their efficacy is limited by HLA restriction, thereby reducing broad applicability (61, 62). In addition, peptide vaccines exhibit low intrinsic immunogenicity and require co-administration with adjuvants, including cytokines or Toll-like receptor (TLR) agonists, to support dendritic cell activation and effective T-cell priming (58, 63).

Synthetic long peptides (SLPs) were developed to overcome these limitations. Because SLPs require intracellular antigen processing, they enable presentation through both MHC class I and class II pathways, thereby promoting coordinated CD4^+^ and CD8^+^ T-cell responses while reducing HLA dependence. ISA101, an SLP-based vaccine comprising overlapping HPV16 E6 and E7 peptides formulated with Montanide ISA-51, demonstrated substantial clinical activity in patients with high-grade vulvar intraepithelial neoplasia. Vaccination induced antigen-specific T-cell responses in all patients and resulted in clinical responses in 79% of patients, including durable complete remission in 9 of 19 patients at two years (64). Notably, the magnitude of CD4^+^ and CD8^+^ T-cell responses correlated with clinical outcome. However, despite the induction of interferon-γ-associated T-cell responses in advanced disease, objective tumor regression was not achieved, highlighting the impact of tumor-mediated immune suppression (65).

These findings have prompted the evaluation of peptide vaccines in combination with immune checkpoint inhibitors and other immunomodulatory approaches. In a phase II study, ISA101 combined with the anti-PD-1 antibody nivolumab demonstrated improved clinical activity in HPV16-positive malignancies. Clinical responses were associated with higher intratumoral infiltration of activated cytotoxic T cells and macrophages, together with enrichment of immune, inflammatory, and interferon-signaling gene signatures (NCT02426892, **Table 1**) (66).

However, a subsequent randomized phase II trial (NCT03669718, **Table 1**) showed that adding ISA101b to cemiplimab did not improve overall response rates in the overall study population, although patients with high PD-L1 expression (CPS ≥20) experienced significant improvements in both overall response rate (ORR) and overall survival (OS) (67). These findings suggest that the clinical efficacy of peptide-based vaccination depends not only on the induction of HPV-specific T-cell responses but also on the characteristics of the tumor immune microenvironment, highlighting the need for predictive biomarkers to identify patients most likely to benefit.

Additional peptide-based platforms have incorporated strategies to enhance immunogenicity. PepCan, which combines HPV16-derived peptides with the Candida-based adjuvant Candin^®^, showed safety and immunogenicity in cervical intraepithelial neoplasia, with histological regression observed in approximately 45% of patients, accompanied by increased TH1 responses and reduced viral load (68, 69). Hespecta (ISA201), which incorporates HPV16 E6 peptides conjugated to the TLR2 ligand Amplivant, has also been investigated in patients with HPV-positive tumors and premalignant lesions (NCT02821494, **Table 1**) (70).

In parallel, short peptide-based vaccines incorporating optimized delivery systems have been developed. Among these, PDS0101 is a lipid-based formulation containing non-MHC-restricted HPV epitopes and the TLR7-activating carrier R-DOTAP and demonstrated a favorable safety profile in a phase I clinical trial (NCT02065973) (71).

This platform is currently being evaluated in a phase II study (NCT04287868, **Table 1**) in combination with bintrafusp alfa (M7824) and NHS-IL12 (PDS01ADC) (72). Bintrafusp alfa is a bifunctional fusion protein that combines PD-L1 blockade with sequestration of transforming growth factor-β (TGF-β) through a TGFβRII “trap,” thereby simultaneously targeting immune checkpoint signaling and TGF-β-mediated immunosuppression within the tumor microenvironment (73, 74). NHS-IL12 is a tumor-targeted IL-12 immunocytokine that localizes to regions of tumor necrosis, promoting TH1-polarized immune responses and enhancing antitumor activity (75). Together, this combination strategy aims to potentiate antigen-specific T-cell responses while overcoming key immunosuppressive mechanisms in the tumor microenvironment (**Figure 2B**).

In contrast to peptide-based vaccines, protein-based vaccines are not restricted by HLA type because they contain the full repertoire of antigenic epitopes. However, they are generally less efficient at inducing cytotoxic T-cell responses and tend to favor humoral immunity due to presentation via MHC class II pathways (62). TA-CIN, a fusion protein comprising HPV16 L2, E6 and E7, induced both CD4^+^ and CD8^+^ T-cell responses in patients with vulvar intraepithelial neoplasia, with complete lesion regression observed in 63% of patients at one year (76). TVGV1, a fusion protein linking HPV16 E7 to a *Pseudomonas aeruginosa* exotoxin domain containing a KDEL endoplasmic reticulum retention signal, was designed to enhance intracellular antigen processing and presentation. In combination with CpG ODN or GPI-0100 adjuvants, TVGV1 induced multifunctional T-cell responses and antitumor activity in preclinical models (77), although clinical evaluation remains ongoing (NCT02576561).

Overall, peptide- and protein-based vaccines consistently induce antigen-specific immune responses but remain limited by insufficient efficacy in established tumors. Advances in computational approaches, including artificial intelligence-driven epitope prediction, are expected to improve identification of immunogenic targets across diverse HLA types and HPV variants, thereby supporting the next generation of therapeutic vaccine design.

### 3.2 DNA-based vaccines

DNA-based vaccines represent an important platform in HPV immunotherapy owing to their favorable tolerability across patient populations, scalable manufacturing with high purity and stability, and their capacity to encode defined antigenic peptides or full-length viral proteins, thereby inducing both humoral and cellular immune responses.

These vaccines employ plasmid vectors to deliver antigen-encoding sequences into host cells, where codon optimization and sequence engineering improve transcriptional efficiency, antigen expression and intracellular trafficking, ultimately enhancing antigen presentation and activation of adaptive immune responses (78, 79). Despite these advantages, DNA vaccines remain limited by low transfection efficiency and relatively modest immunogenicity.

To improve DNA uptake and immunogenicity, advanced delivery approaches such as electroporation have been developed. Electroporation transiently increases cell membrane permeability, facilitating intracellular DNA delivery while simultaneously inducing localized inflammation that recruits antigen-presenting cells (APCs) to the vaccination site (80). Alternatively, gene gun-mediated delivery propels DNA-coated gold particles into the epidermis, enabling efficient transfection of dermal dendritic cells (DCs), which subsequently migrate to lymphoid organs for T-cell priming (81).

In parallel with advances in DNA delivery, antigen engineering strategies have been developed to optimize antigen processing and presentation, thereby enhancing HPV-specific T-cell responses. HPV16 E7 has been fused to MHC class I-targeting proteins and domains, including *Mycobacterium tuberculosis* HSP70 (82), γ-tubulin (83), FMS-like tyrosine kinase 3 ligand (Flt3L) (84), and the translocation domain of *Pseudomonas aeruginosa* exotoxin A (ETA(dII)) (85).

DNA vaccines incorporating E7 linked to calreticulin (CRT), a calcium-binding chaperone involved in antigen processing, significantly improved MHC class I antigen presentation and elicited robust E7-specific CD8^+^ T-cell responses (86). In parallel, fusion of antigenic sequences to an endoplasmic reticulum signal peptide and the lysosomal targeting domain of lysosome-associated membrane protein 1 (LAMP-1) enhanced MHC class II presentation and promoted CD4^+^ T-cell responses (87).

Clinical translation of therapeutic HPV DNA vaccines also requires careful consideration of safety. Because E7 is intrinsically oncogenic through its interaction with the retinoblastoma (Rb) protein, genetically attenuated variants incorporating mutations at positions 24 and/or 26 were developed to disrupt Rb binding and abolish transforming potential (88).Vector selection is likewise critical for regulatory compliance. The pcDNA3 plasmid contains an ampicillin resistance gene, which may limit its suitability for human application. By contrast, the pNGVL4a vector, derived from the clinically tested pNGVL-3 backbone, lacks this resistance cassette and has therefore been adopted for clinical-grade DNA vaccine development (89).

Several therapeutic HPV DNA vaccines have subsequently advanced to clinical evaluation, including GX-188E incorporating Flt3L (90, 91), pNGVL4a-CRT/E7(detox) (92), pNGVL4a-Sig/E7(detox)/HSP70 (93), VGX-3100 (94) and MEDI0457 (95). MEDI0457 is based on the VGX-3100 platform but additionally incorporates a plasmid encoding interleukin-12 to enhance cellular immunity. Clinical efficacy has varied across platforms. GX-188E demonstrated lesion regression in approximately 67% of patients in phase I/II studies, whereas VGX-3100 achieved histopathological regression in nearly half of treated patients compared with placebo (94). Other constructs demonstrated response rates of approximately 30% (92, 93).

Given the risk of recurrence following surgical excision of cervical lesions, viral clearance represents a critical endpoint in therapeutic vaccination (96). VGX-3100, GX-188E, and MEDI0457 demonstrated correlations between HPV DNA clearance and histopathological regression (90, 94, 95), whereas pNGVL4a-CRT/E7 (detox) did not consistently reduce viral load (92). DNA vaccines have also been evaluated in combination with immune checkpoint inhibitors, including pembrolizumab, durvalumab and atezolizumab, in clinical trials in HPV-associated malignancies (**Table 1**).

### 3.3 RNA-based vaccines

The emergence of mRNA vaccines has substantially advanced vaccine technology by enabling rapid and flexible antigen expression without the risk of host genomic integration (97). mRNA molecules can also serve as intrinsic adjuvants through activation of innate immune receptors such as TLR7 and TLR8 expressed on dendritic cells (98).

However, the intrinsic instability of naked mRNA and its poor delivery efficiency initially limited clinical translation. Consequently, mRNA is commonly formulated with delivery systems, including protamine complexes, cationic lipids, polymers and lipid nanoparticles (LNPs), which protect against enzymatic degradation and facilitate cytosolic delivery (99–102).

Preclinical studies demonstrated that intravenous administration of liposome-formulated HPV16 E7 mRNA (RNA-lipoplex, RNA-LPX) selectively targets dendritic cells in lymphoid tissues, inducing potent antigen-specific CD8^+^ T-cell responses capable of mediating tumor regression in murine HPV-positive models (103). Clinical translation has progressed with the BioNTech candidate BNT113 encoding HPV16 E6 and E7. Early clinical evaluation in the phase I HARE-40 trial (NCT03418480) demonstrated induction of E6/E7-specific CD8^+^ T-cell responses and anti-E7 antibodies in patients with advanced HPV16-positive malignancies (104). A phase II study (NCT04534205, **Table 1**) is currently evaluating BNT113 in combination with pembrolizumab for recurrent or metastatic HPV16-positive head and neck squamous cell carcinoma (105).

To expand antigenic targeting beyond E6 and E7, the mHTV-03E2 vaccine incorporates shuffled HPV16/18 E2, E6, and E7 antigens fused to a tissue plasminogen activator (tPA) signal peptide and Flt3 ligand to enhance antigen presentation. As E2 is expressed early during HPV infection and regulates viral replication, its inclusion may broaden immune targeting across different stages of the HPV life cycle. In preclinical models, vaccination induced robust E6/E7-specific cellular immunity together with E2-specific humoral responses and promoted effector-memory CD8^+^ T-cell differentiation, while demonstrating synergistic antitumor activity in combination with PD-L1 blockade (106). Similarly, the E7-Trimix platform co-expresses the immunostimulatory molecules CD40L, constitutively active TLR4, and CD70 together with HPV antigens to enhance dendritic-cell activation and co-stimulatory signaling, resulting in increased tumor-infiltrating antigen-specific CD8^+^ T cells and suppression of tumor growth in preclinical models (107).

Beyond conventional non-replicating mRNA vaccines, next-generation RNA platforms and engineering strategies have focused on improving antigen expression, stability, and immunogenicity. Self-amplifying RNA (saRNA) is derived from alphavirus replicons, in which the structural protein genes are replaced by the target antigen while the nonstructural proteins required for RNA replication are retained, enabling intracellular RNA amplification and prolonged antigen expression at substantially lower doses than conventional mRNA (108). In HPV-associated tumor models, a Venezuelan equine encephalitis (VEE)-based saRNA vaccine encoding either a modified HPV16 E7 antigen or a GM-CSF-E7 fusion protein and formulated using liposome-protamine-RNA (LPR) nanoparticles or lipid nanoparticles (LNPs) was evaluated. Compared with the saRNA vaccine encoding E7 alone, the LPR-saRNA-GM-CSF-E7 formulation enhanced antigen-presenting cell activation, promoted E7-specific CD8^+^ T-cell cytotoxic responses, and reshaped the tumor microenvironment by reducing immunosuppressive cell populations and increasing Th1-associated cytokine expression, resulting in delayed tumor progression and prolonged survival in TC-1 tumor-bearing mice (109).

Another next-generation RNA platform is circular RNA (circRNA), whose covalently closed structure confers greater resistance to exonuclease degradation and enables prolonged antigen expression compared with conventional linear mRNA. An LNP-formulated circRNA vaccine encoding engineered HPV16 E6/E7 antigens fused to a calreticulin (CRT) signal sequence was developed to enhance antigen processing and MHC class I presentation. In preclinical HPV16 tumor models, this vaccine elicited robust antigen-specific CD8^+^ T-cell responses, induced durable memory immunity, achieved complete tumor regression with prolonged survival, and further improved therapeutic efficacy when combined with PD-L1 blockade (110).

Therapeutic mRNA vaccine engineering continues to evolve. Computational sequence optimization has emerged as a strategy to improve mRNA design by optimizing RNA secondary structure and codon usage, thereby enhancing mRNA stability, translational efficiency, protein expression, and immunogenicity (111). Zhang et al. developed MTS107, an LNP-encapsulated therapeutic mRNA vaccine encoding mutated HPV16/18 E6/E7 antigens together with GM-CSF. The optimized MLX0487–23 lipid nanoparticle, selected from six candidate formulations based on superior antitumor activity, preferentially delivered the mRNA to dendritic cells and macrophages, particularly within the spleen, facilitating antigen expression and immune activation. In HPV16- and HPV18-positive syngeneic tumor models, MTS107 induced HPV-specific CD8^+^ T-cell responses, dose-dependent tumor growth inhibition, and prolonged survival. Combination with anti-PD-1 checkpoint blockade achieved complete tumor remission, while co-administration of an OX40L-encoding mRNA further enhanced antitumor efficacy compared with the GM-CSF-encoding vaccine alone (112).

Despite encouraging preclinical findings and promising early clinical outcomes, the therapeutic efficacy of RNA-based vaccines for HPV-associated malignancies remains to be established in larger randomized clinical trials.

### 3.4 Live vector-based vaccines

Live vector–based vaccines employ bacterial or viral carriers to deliver antigens directly to antigen-presenting cells. These vectors are inherently immunogenic owing to their pathogen-associated molecular patterns (PAMPs) (113). However, the use of live vectors raises safety concerns; therefore, most vaccine vectors are attenuated or genetically modified to limit replication. In addition, pre-existing immunity against the vector may lead to rapid clearance by the host immune system, potentially reducing vaccine efficacy (114, 115).

#### 3.4.1 Bacterial vectors

Several bacterial species, including *Listeria monocytogenes*, *Lactococcus lactis*, *Lactobacillus plantarum* and *Lactobacillus casei*, have been explored as live vectors for therapeutic HPV vaccines (116–120). Among these, *Listeria monocytogenes* has emerged as a particularly promising platform because of its ability to infect phagocytic cells and replicate intracellularly. Following phagocytic uptake, *Listeria* secretes the pore-forming toxin listeriolysin O (LLO), which enables escape from the phagosome and subsequent replication within the cytoplasm of infected cells (121). This intracellular localization allows bacterial antigens to access both cytosolic and endosomal processing pathways, enabling presentation through MHC class I and class II molecules and activation of CD8^+^ cytotoxic and CD4^+^ helper T-cell responses (122). One of the most extensively studied *Listeria*-based HPV vaccines is axalimogene filolisbac (ADXS11-001), an attenuated *L. monocytogenes* strain engineered to secrete a fusion protein consisting of truncated LLO linked to the HPV16 E7 antigen (123).

In a randomized phase II trial in recurrent or refractory cervical cancer, ADXS11-001, administered alone or in combination with cisplatin, demonstrated a pooled overall response rate of 15.9% and a 12-month overall survival rate of 34.9%, with no additional benefit observed from the addition of cisplatin (124). Similarly, in a small phase I/II study of locally advanced anal cancer, ADXS11–001 combined with standard chemoradiotherapy was associated with complete clinical responses in all evaluable patients, and 89% of patients remained progression-free after a median follow-up of 42 months (125). However, because neither study included a standard-of-care control arm, the efficacy of ADXS11–001 relative to standard therapy remains uncertain. ADXS11–001 has generally shown an acceptable safety profile, although rare serious adverse events, including systemic listeriosis, have been reported (NCT01598792) (126).

Other bacterial vectors have also been investigated for therapeutic HPV vaccination. *Lactobacillus casei* can be administered non-invasively, typically via oral or intranasal routes, and is considered relatively safe because it lacks endotoxin. A phase I/IIa clinical study evaluating an oral *L. casei*–based vaccine expressing HPV16 E7 (GLBL101c, **Table 1**) in patients with HPV16-positive CIN3 demonstrated induction of E7-specific mucosal cellular immune responses and histological regression of lesions from CIN3 to CIN2 in approximately 70% of patients. As this was a single-arm study without a placebo or observation control group, the contribution of vaccination to the observed regression cannot be established. Nevertheless, the authors noted that the observed regression rate exceeded the approximately 10% spontaneous CIN3-to-CIN2 regression rate estimated from previous non-intervention cohorts and placebo arms of HPV therapeutic vaccine trials, although randomized placebo-controlled studies are required to confirm the clinical efficacy of GLBL101c. No significant adverse events were reported, and the presence of E7-specific lymphocytes in cervical tissue correlated with pathological regression (127).

#### 3.4.2 Viral vectors

Vaccinia virus-based vectors are widely used in therapeutic HPV vaccination because their genetic stability and large cloning capacity enable efficient insertion and expression of transgenic DNA. Tipapkinogen sovacivec (TS; TG4001) is a replication-deficient modified vaccinia Ankara (MVA) vector encoding HPV16 E6 and E7 together with IL-2. In a phase II study (NCT01022346) involving patients with CIN2/3, TG4001 induced lesion regression during 2.5 years of follow-up, independent of high-risk HPV type (128).

TG4001 is currently being evaluated in combination with the PD-L1 inhibitor avelumab in patients with HPV16-positive recurrent or metastatic cancers (NCT03260023, **Table 1**), where early-phase studies showed acceptable safety and preliminary antitumor activity (129).

Another MVA-based therapeutic vaccine targeting HPV16 E2 has also demonstrated encouraging clinical activity. In a phase III study including 1,356 patients with HPV-associated anogenital intraepithelial lesions, histologic regression was observed in 89% of women and 100% of men, with HPV DNA clearance observed in 83% of treated individuals. These findings are consistent with the role of E2 in downregulating E6 and E7 expression and promoting apoptosis (130).

TA-HPV, a live recombinant vaccinia virus encoding modified HPV16/18 E6 and E7 antigens, has been evaluated in heterologous DNA prime–viral boost regimens. In a phase I study in patients with HPV16-positive CIN2/3 (NCT00788164), priming with an E7-targeted DNA vaccine (pNGVL4a-Sig/E7(detox)/HSP70) followed by a TA-HPV boost was well tolerated and induced HPV-specific CD8^+^ T-cell responses (131, 132).

Human adenoviruses represent another widely used viral vector system because of their high transduction efficiency and broad cellular tropism, enabling gene delivery to both dividing and non-dividing cells. However, pre-existing immunity to common adenoviral serotypes may limit therapeutic efficacy (133). To overcome this limitation, less prevalent serotypes such as Ad26 and Ad35 have been explored for delivery of HPV antigens, including E2, E6 and E7 fusion constructs. These vectors demonstrated strong immunogenicity and antitumor activity in preclinical models (134). A heterologous prime–boost strategy using intramuscular priming followed by intravaginal boosting with Ad26- and Ad35-based vectors induced HPV-specific CD8^+^ T cells producing IFN-γ and TNF-α in the cervicovaginal mucosa, supporting their potential for persistent HPV infection and premalignant disease (135).

To further overcome pre-existing immunity, non-human primate adenoviral vectors have been developed. A chimpanzee adenovirus vector (ChAdOx1) encoding conserved HPV regions induced strong and durable T-cell responses, particularly when combined with MVA in prime–boost regimens. This strategy generated polyfunctional T cells capable of trafficking to cervical tissue (136). Similarly, a gorilla adenovirus–based vector (PRGN-2009), incorporating multiple HPV16 and HPV18 E6/E7 epitopes, has shown promising preclinical activity and is currently under clinical evaluation (NCT04432597) (137, 138).

RNA virus–based vectors represent an emerging platform, particularly those derived from alphaviruses and arenaviruses. Replicon-based systems enable sustained antigen expression without genomic integration, allowing repeated administration (139). The Semliki Forest virus (SFV)–based vaccine Vvax001, encoding HPV16 E6 and E7, has demonstrated clinical activity and favorable tolerability in patients with HPV16-positive CIN3 in a phase I/II study (NCT03141463, **Table 1**) (140).

Additional RNA-based platforms are advancing in clinical development. Arenavirus-derived vectors, including HB-201 and HB-202, based on lymphocytic choriomeningitis virus and Pichinde virus, respectively, are being evaluated in a phase I/II study (NCT04180215, **Table 1**), both as monotherapy and in combination with immune checkpoint blockade. Preliminary data indicate that these vectors promote robust pro-inflammatory cytokine production and expansion of HPV-specific CD8^+^ T cells. Alternating administration of HB-201 and HB-202 enhanced these responses, with HPV-specific CD8^+^ T cells accounting for up to 40% of the circulating CD8^+^ T-cell population (141).

### 3.5 Dendritic cell-based vaccines

Dendritic cells (DCs) play a central role in initiating both innate and adaptive immune responses, making them attractive targets for therapeutic vaccine development. Through antigen presentation via MHC class II and class I pathways, DCs activate antigen-specific CD4^+^ and CD8^+^ T cells (142). Therapeutic DC vaccines typically involve generation of autologous DCs from patients followed by ex vivo loading with HPV-derived antigens, either through pulsing with HPV-specific peptide or protein antigens or through genetic modification using DNA or viral vectors encoding viral oncoproteins such as E6 and E7 prior to reinfusion to stimulate antigen-specific immunity (142, 143).

Early clinical studies showed that DC vaccination was feasible, well tolerated and capable of inducing HPV-specific T-cell responses. In patients with early-stage cervical cancer, vaccination with DCs pulsed with HPV16/18 E7 antigens and administered together with keyhole limpet hemocyanin elicited detectable CD4^+^ and CD8^+^ T-cell responses with minimal toxicity (144). Despite this immune activation, clinical benefit has generally been limited, partly because of tumor immune-evasion mechanisms such as HLA downregulation (145, 146). In a randomized phase I trial, vaccination with tumor lysate–primed DCs resulted in a complete clinical response in one patient (147). Alternative strategies have been explored, including pre-immune dendritic cells (PIDCs), which lack full co-stimulatory activity but are easier to generate and have been shown to induce HPV-specific immunity in subsets of patients (148).

The broader clinical application of DC vaccines remains restricted by several practical and biological limitations. Generation of patient-specific DC products requires individualized ex vivo cell manufacturing that is labor-intensive, costly and difficult to scale. Additional challenges include HLA restriction, variability in vaccine quality arising from differences in culture protocols and the limited lifespan of transferred DCs (58, 59, 149, 150). Approaches to prolong DC survival have therefore been explored; for example, short interfering RNA (siRNA) targeting pro-apoptotic pathways enhanced E7-specific CD8^+^ T-cell activation and antitumor activity in preclinical models (151, 152).

### 3.6 B cell-based vaccines

BVAC-C is an antigen-presenting cell–based vaccine composed of autologous B cells and monocytes engineered to present HPV16/18 E6/E7 antigens and loaded with the invariant NKT-cell ligand α-galactosylceramide (α-GalCer) (**Figure 3A**) (153). These cell populations are abundant in peripheral blood and can be readily collected by apheresis, which simplifies manufacturing and offers a practical advantage for clinical use. BVAC-C activates HPV-specific T cells as well as NKT and NK cells and may overcome immune-evasion mechanisms such as loss of MHC class I expression and effector-cell dysfunction within the tumor microenvironment (154). These immunological properties provided the rationale for subsequent clinical evaluation of BVAC-C in patients with HPV-associated malignancies. Early clinical studies showed a favorable safety profile in recurrent cervical cancer, with no dose-limiting toxicities observed in the phase I setting (NCT02866006, **Table 1**) (155). In phase II evaluation, objective responses were observed in 19.2% of patients and disease control in 53.8%. Ongoing studies are exploring BVAC-C in combination with PD-L1 inhibitors (156).

**Figure 3:**
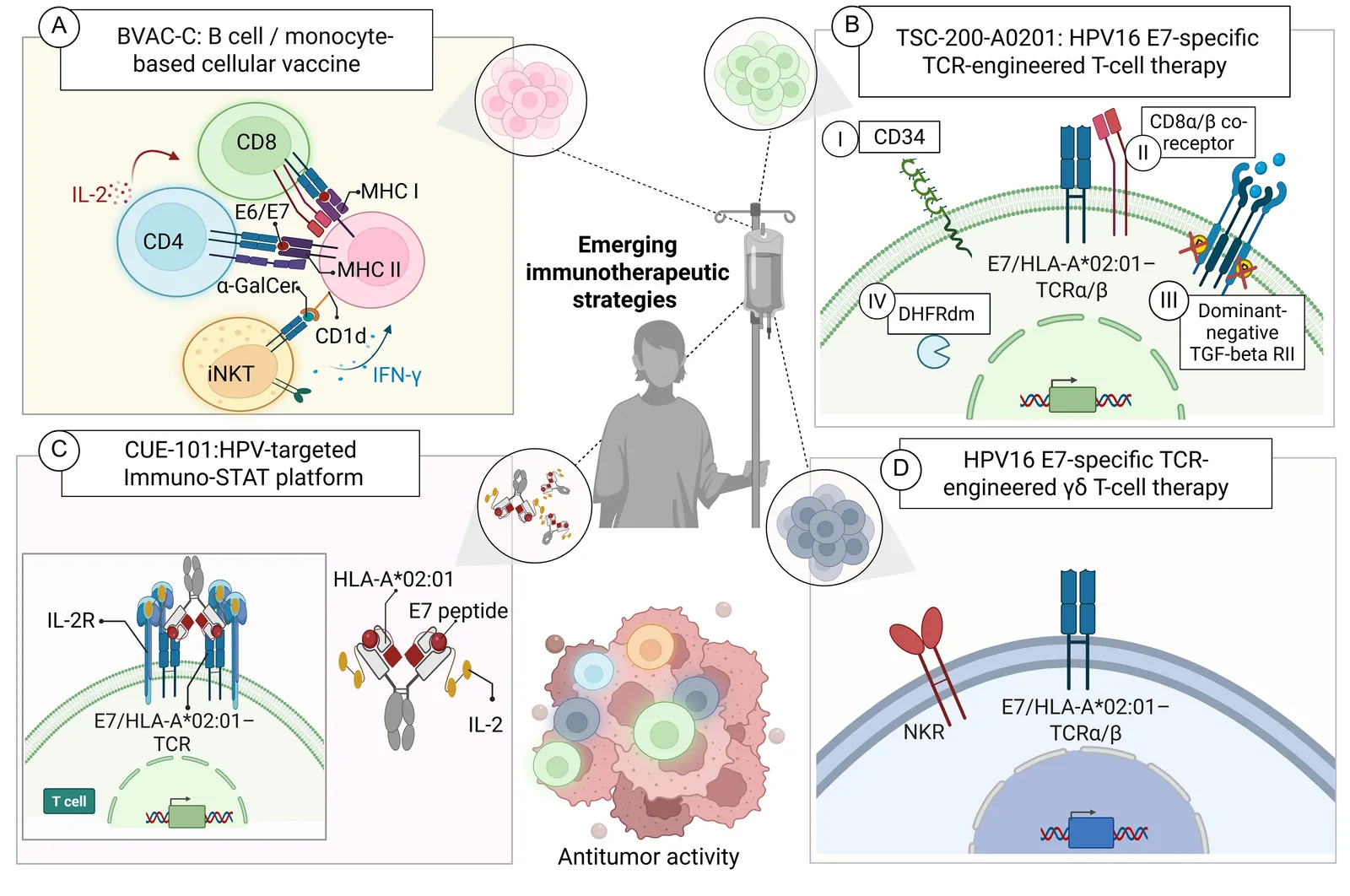
Emerging immunotherapeutic strategies for HPV-associated malignancies. **(A)** BVAC-C, a B cell/monocyte-based vaccine platform presenting HPV16/18 E6/E7 antigens and incorporating α-galactosylceramide (α-GalCer) to induce coordinated activation of HPV-specific T cells and iNKT cells. **(B)** TSC-200-A0201, a T-cell therapy engineered to express an HLA-A*02:01-restricted TCR targeting HPV16 E7 together with CD8α/β co-receptors, a dominant-negative TGF-β receptor II to enhance resistance to immunosuppressive signaling, a CD34 tag for cell tracking, and a mutated dihydrofolate reductase (DHFRdm) selection marker. **(C)** CUE-101 (Immuno-STATs), a modular fusion protein comprising two HPV16 E7 peptide–HLA-A*02:01 complexes linked to four affinity-attenuated IL-2 molecules and an effector-attenuated IgG1 Fc domain, designed to selectively activate tumor-specific T cells through coupled antigen-specific TCR engagement and targeted cytokine delivery. **(D)** γδ T-cell–based therapy engineered to express an HLA-A*02:01-restricted TCR targeting HPV16 E7 while retaining endogenous NKR-mediated cytotoxicity, enabling recognition of HLA-deficient tumor cells.

## 4 Adoptive T cell therapy

Adoptive T-cell therapy (ACT) uses autologous T lymphocytes that undergo ex vivo expansion or genetic modification to enhance tumor recognition before reinfusion into the patient. Unlike prophylactic or therapeutic vaccines, ACT delivers tumor-reactive T cells directly, bypassing endogenous antigen-presenting cell priming (157, 158). In HPV-associated malignancies, ACT has primarily focused on targeting the viral oncoproteins E6 and E7, which are constitutively expressed and represent non-self-antigens (159). Tumor-infiltrating lymphocyte (TIL) therapy enriched for E6/E7-reactive T cells has shown clinical activity in metastatic cervical cancer; in a phase II study (NCT01585428, **Table 1**), objective responses were observed in 3 of 9 patients, including two complete remissions and one partial response (160). However, because cervical tumors co-express non-viral tumor-associated antigens, the precise contribution of HPV-specific T-cell recognition to tumor regression remains uncertain. In addition, TIL therapy is resource-intensive and its efficacy is constrained by the immunosuppressive tumor microenvironment. Cytokine-induced killer (CIK) cell therapy provides a technically simpler alternative through expansion of peripheral blood mononuclear cells rather than tumor-derived lymphocytes. In cervical cancer, CIK therapy combined with chemoradiotherapy showed short-term clinical benefit, warranting further investigation (161). A more precisely targeted strategy is TCR-engineered T-cell therapy, in which autologous T cells are genetically modified to express receptors specific for HPV-derived peptides presented by MHC molecules rather than expanding naturally occurring tumor-reactive clones. Therapeutic activity depends on TCR affinity and adequate surface expression of functional α/β heterodimers; approaches including promoter optimization and engineering of TCR chains are being explored to improve antitumor potency (157, 162).

Clinical translation has largely focused on HLA-A*02:01-restricted HPV16 epitopes. In a phase I/II trial (NCT02280811, **Table 1**), adoptive transfer of autologous T cells engineered to recognize the HLA-A*02:01-restricted E6_29–38_ epitope mediated objective tumor regression in patients with HPV16-positive epithelial malignancies. Of 12 treated patients, two with anal carcinoma experienced partial responses. In one patient, a pulmonary metastasis completely regressed and two additional lesions decreased in size, enabling surgical resection; the patient remained free of detectable disease three years after infusion. Correlative analyses revealed intratumoral accumulation of E6-specific T cells with elevated PD-1 signaling, consistent with checkpoint-mediated functional attenuation within the tumor microenvironment. By contrast, non-responding tumors exhibited features of immune resistance, including a truncating mutation in IFNGR1 impairing interferon-γ signaling and loss of HLA-A*02:01 expression (163).

Similarly, a phase I trial targeting the HLA-A*02:01-restricted E7_11–19_ epitope (NCT02858310, **Table 1**) reported objective responses in 6 of 12 patients with metastatic HPV-associated epithelial cancers, including three complete regressions of metastatic lesions, with several responses sustained for months. The comparatively greater activity of E7-directed constructs has been attributed to higher functional avidity, prolonged peptide–MHC stability and improved transduction efficiency (164, 165). Transcriptomic analyses of non-responding tumors revealed alterations consistent with those observed in E6-directed therapy (166). These findings prompted initiation of a randomized phase II clinical trial (NCT05686226).

Overall, TCR-engineered T-cell therapy has shown clear clinical activity in HPV-associated cancers. However, durable clinical benefit remains constrained by tumor-intrinsic immune escape and an immunosuppressive tumor microenvironment, highlighting the need for rational combination strategies that restore antigen processing and sustain T-cell function.

Ongoing efforts aim to expand the repertoire of TCRs specific for HPV16 E6 and E7 oncoproteins across diverse HLA types to improve patient coverage and reduce immune escape (167). Two novel HLA-A*11:01-restricted HPV16 epitopes, E6_93–101_ (TTLEQQYNK; TTL) and E7_89–97_ (IVCPICSQK; IVC), were identified through combined *in silico* prediction and experimental validation. HLA-A*11:01 is among the most prevalent HLA alleles globally, particularly in Asian populations (168). TCR-engineered T cells specific for TTL and IVC efficiently recognized and eliminated HLA-A*11:01-positive, E6/E7-expressing tumor cells *in vitro* and *in vivo*, supporting their potential application in immunotherapy for HLA-A*11:01-positive patients (169).

Next-generation T-cell therapies targeting HPV E6 and E7 oncoproteins are advancing rapidly, with several approaches currently under development. TSC-200-A0201 employs a naturally derived high-avidity TCR specific for HPV16 E7 presented by HLA-A*02:01 (**Figure 3B**). This construct incorporates co-expression of CD8α/β co-receptors, a dominant-negative TGFβ receptor II to resist immunosuppressive signaling, a CD34 epitope tag for cell tracking and a mutated dihydrofolate reductase for selective expansion during manufacturing. Preclinical studies showed potent antigen-dependent cytotoxicity, cytokine secretion and proliferation of both CD4^+^ and CD8^+^ T cells while maintaining activity in the presence of physiological levels of TGFβ. *In vivo* models demonstrated effective tumor control without detectable off-target toxicity against a broad panel of healthy human cells. These preclinical findings supported FDA clearance for clinical development of TSC-200-A0201 under the T-Plex phase I master protocol for HPV16-positive solid tumors (170).

γδ T cells have recently attracted considerable interest for adoptive therapy in HPV-associated malignancies. Unlike conventional αβ T cells, they recognize targets in an MHC-independent manner, reducing the risk of graft-versus-host disease. Gamma Delta One T cells (DOTs) have been isolated, expanded and engineered to express HPV16 E7-specific TCRs with transduction efficiencies comparable to αβ T cells. *In vitro*, engineered DOTs exhibited potent cytotoxicity against HLA-A*02:01-positive tumors and superior killing of HLA class I-deficient variants relative to conventional αβ TCR-T cells. This enhanced activity has been attributed to dual targeting mediated by antigen-specific TCR recognition together with innate-like cytotoxicity through NK receptors (NKR) such as NKG2D, DNAM-1 and NKp30, enabling recognition of tumors with HLA downregulation (**Figure 3D**). These properties support the potential application of γδ T cells in off-the-shelf TCR-engineered therapies for HPV-associated cancers (171, 172).

### 4.1 Immuno-STATs

Immuno-STATs (Selective Targeting and Alteration of T cells) represent a distinct class of T-cell-directed immunotherapies designed to selectively activate tumor-specific T cells *in vivo* without *ex vivo* cell manipulation. These modular fusion proteins combine a tumor-associated peptide–HLA complex with affinity-attenuated IL-2, coupling antigen-specific T-cell receptor engagement to a proliferative cytokine signal while limiting the toxicity associated with non-targeted IL-2 therapy (173). CUE-101 was the first Immuno-STAT to enter clinical evaluation (**Figure 3C**) (174). In a phase I study involving patients with HPV16-positive recurrent or metastatic head and neck squamous cell carcinoma (NCT03978689, **Table 1**), CUE-101 showed a favorable safety profile and preliminary clinical activity both as monotherapy and in combination with pembrolizumab (175–177).

## 5 Conclusions and perspectives

HPV-associated carcinogenesis is driven by the viral oncoproteins E6 and E7, which disrupt tumor suppressor pathways, sustain proliferation, and promote invasion and disease progression. Their continuous expression and essential role in tumor maintenance make them compelling therapeutic targets.

Current immunotherapeutic strategies, including therapeutic vaccines and adoptive T-cell therapies, aim to induce antigen-specific T-cell responses against these oncoproteins. Despite encouraging immunological activity, clinical efficacy has remained variable across studies, indicating that induction of HPV-specific T-cell responses alone is insufficient to achieve durable clinical benefit. Instead, therapeutic success depends on the quality, persistence, and functional capacity of HPV-specific T cells within the tumor microenvironment.

A major limiting factor is the immunosuppressive tumor microenvironment, which restricts T-cell activity through inhibitory cytokines and suppressive immune populations such as tumor-associated myeloid cells (178, 179). Immune escape through impaired antigen presentation, altered interferon signaling, and increased PD-1 expression on tumor-infiltrating HPV16 E6-specific T cells has also emerged as an important mechanism of resistance in TCR-engineered T-cell studies.

The consistent disconnect between immunogenicity and clinical efficacy observed across single-agent platforms has shifted the field toward rational combination strategies that simultaneously enhance antigen-specific immunity and overcome tumor-mediated immune suppression. These strategies include combining therapeutic vaccination with immune checkpoint blockade or tumor-targeted cytokines to sustain intratumoral T-cell function, integrating vaccination with chemotherapy or radiotherapy to promote immunogenic cell death and antigen cross-priming, and combining vaccination with adoptive T-cell therapies to improve the expansion, persistence, and effector function of transferred HPV-specific T cells (72, 180–182). Heterologous prime–boost strategies that combine different vaccine platforms can also overcome platform-specific limitations, reduce the impact of vector-specific immunity, and enhance the magnitude and durability of antigen-specific immune responses (183).

Beyond immune-based approaches, gene-targeting strategies are also emerging as a therapeutic direction. Approaches based on RNA interference and genome editing, including CRISPR/Cas9, aim to directly disrupt HPV oncogene expression within tumor cells. Preclinical studies indicate that these approaches can induce apoptosis, inhibit proliferation, and reduce tumorigenicity in HPV-positive models, while early-phase clinical trials are beginning to evaluate their safety and feasibility in precancerous lesions (NCT02800369; NCT03057912). Although mechanistically distinct from immunotherapy, these strategies may complement immune-mediated therapies by reducing tumor burden and enhancing tumor susceptibility to immune-mediated clearance (184–189).

Despite these advances, several challenges remain. Interpretation of clinical efficacy across therapeutic platforms is complicated by substantial differences in disease stage, patient populations, treatment combinations, and clinical endpoints, together with the limited number of randomized studies currently available. In addition, HLA restriction, anti-vector immunity, and an incomplete understanding of resistance mechanisms continue to constrain therapeutic efficacy. Reliable predictive biomarkers also remain lacking, as currently available markers, including PD-L1 expression, provide only modest precision for patient stratification (190). Future studies should therefore prioritize the validation of biomarkers that accurately predict therapeutic response and guide patient selection across different immunotherapeutic platforms. Integrating virological endpoints, such as HPV DNA clearance, alongside immunological and clinical outcome measures may also provide a more comprehensive assessment of therapeutic benefit.

Beyond improving therapeutic efficacy, practical considerations—including manufacturing complexity, scalability, storage requirements, and treatment cost—remain critical for the successful clinical translation of therapeutic HPV immunotherapies (191). These factors are particularly important for low- and middle-income countries, where the burden of HPV-associated malignancies remains high and access to complex individualized therapies may be limited (192). Future therapeutic platforms should therefore be evaluated not only for their clinical efficacy but also for their feasibility for broader clinical implementation.

Overall, the field is moving toward integrated therapeutic paradigms that combine antigen-specific targeting with modulation of the tumor microenvironment and, potentially, direct targeting of viral oncogenes. Together, these advances provide a strong foundation for the development of next-generation HPV-specific therapies aimed at improving outcomes for patients with HPV-associated malignancies.
